# Three-gene risk model in papillary renal cell carcinoma: a robust likelihood-based survival analysis

**DOI:** 10.18632/aging.104001

**Published:** 2020-11-05

**Authors:** Yutao Wang, Kexin Yan, Jiaxing Lin, Jianfeng Wang, Zhenhua Zheng, Xinxin Li, Zhixiong Hua, Yuepeng Bu, Jianxiu Shi, Siqing Sun, Xuejie Li, Yang Liu, Jianbin Bi

**Affiliations:** 1 Department of Urology, The First Hospital of China Medical University, Shenyang 110001, Liaoning, China; 2 Department of Dermatology, The First Hospital of China Medical University, Shenyang 110001, Liaoning, China; 3 Joint Fund of Science and Technology Department of Liaoning Province and State Key Laboratory of Robotics, Shenyang 110001, Liaoning, China

**Keywords:** papillary renal cell carcinoma (PRCC), robust risk model, weighted gene co-expression network analysis (WGCNA), immune infiltration, tumor mutation burden (TMB)

## Abstract

Background: Papillary renal cell carcinoma (PRCC) accounts for 15% of all renal cell carcinomas. The molecular mechanisms of renal papillary cell carcinoma remain unclear, and treatments for advanced disease are limited.

Result: We built the computing model as follows: Risk score = 1.806 * *TPX2* - 0.355 * *TXNRD2* - 0.805 * *SLC6A20*. The 3-year AUC of overall survival was 0.917 in the training set (147 PRCC samples) and 0.760 in the test set (142 PRCC samples). Based on the robust model, M2 macrophages showed positive correlation with risk score, while M1 macrophages were the opposite. PRCC patients with low risk score showed higher tumor mutation burden. *TPX2* is a risk factor, and co-expression factors were enriched in cell proliferation and cancer-related pathways. Finally, the proliferation and invasion of PRCC cell line were decreased in the *TPX2* reduced group, and the differential expression was identified. *TPX2* is a potential risk biomarker which involved in cell proliferation in PRCC.

Conclusion: We conducted a study to develop a three gene model for predicting prognosis in patients with papillary renal cell carcinoma. Our findings may provide candidate biomarkers for prognosis that have important implications for understanding the therapeutic targets of papillary renal cell carcinoma.

Method: Gene expression matrix and clinical data were obtained from TCGA (The Cancer Genome Atlas), GSE26574, GSE2048, and GSE7023. Prognostic factors were identified using “survival” and “rbsurv” packages, and a risk score was constructed using Multivariate Cox regression analysis. The co-expression networks of the factors in model were constructed using the “WGCNA” package. The co-expression genes of factors were enriched and displayed the biological process. Based on this robust risk model, immune cells infiltration proportions and tumor mutation burdens were compared between risk groups. Subsequently, using the PRCC cell line, the role of TPX2 was determined by Cell proliferation assay, 5-Ethynyl-20-deoxyuridine assay and Transwell assay.

## INTRODUCTION

Renal cell carcinoma accounts for 2–3% of all cancers. In recent years, the associated incidence and mortality have increased [1]. Renal cancer includes various tissue types, characterized by various genetic driving factors [2]. Papillary renal cell carcinoma (PRCC), which accounts for 15–20% of renal cancer, is a heterogeneous disease, divided into two subtypes, PRCC1 and PRCC2 [3]. Compared with clear cell renal cell carcinoma (ccRCC), the occurrence of organ-defined tumors (pT1-2N0M0) of PRCC and the five-year tumor-specific survival rate are higher [4]. There is no effective treatment for patients with advanced papillary renal cell carcinoma. Focusing on the molecular mechanisms of PRCC occurrence and development will help to identify candidate biomarkers and therapeutic targets.

With the development of high-throughput sequencing technology, we obtained a disease expression matrix from open source databases. At present, the predictive model method acts as effective method to find the key prognosis factors. Cao et al. constructed a recurrence prediction model based on 17 prognosis-related protein coding genes [5]. Zhang et al. constructed a prognostic survival model based on 17 mutation genes [6]. Klatte et al. constructed a VENUSS prognostic model to predict disease recurrence for non-metastatic papillary renal cell carcinoma, which VENUSS contained four clinical indicators (VEnous extension, NUclear grade, Size, Stage) score [7]. Gao et al. constructed a prognostic score model composed of five protein-editing genes [8]. Lee et al. updated the Leibovich score to obtain more accurate results for mortality and prognosis of renal cell carcinoma [9]. These studies assessed prognostic status by establishing prognostic scoring systems and assigning risk scores to each patient. After external data verification, they achieved good predictive power. These findings suggest that it is critical to mine key prognostic factors to establishing prognostic prediction models.

In this paper, by constructing a prognostic prediction model in The Cancer Genome Atlas (TCGA), we identified key risk factors and protection factors of the disease. By establishing the prognostic co-expression network of key factors, we clarified function of the co-expression network and the related pathways. Finally, we explored the correlation between cancer-related immune processes, tumor mutation burden and risk score, and determined the important biological significance of risk factors.

## RESULTS

### Prognosis factors in TCGA

**Figure 1:**
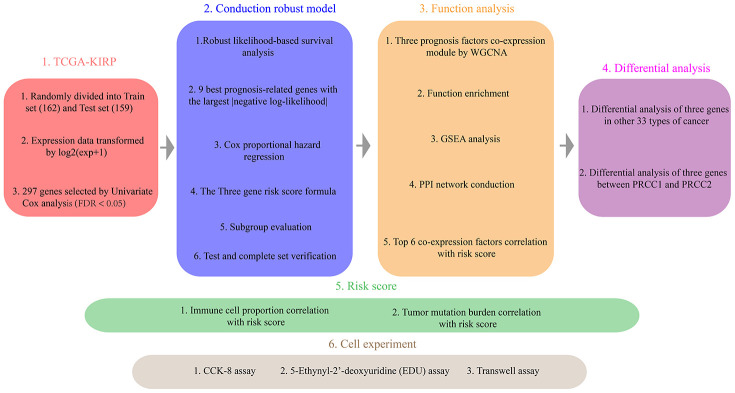
**The Overview of the strategy.**

The Overview of the strategy was shown in Figure 1. We obtained 289 samples of papillary renal cell carcinoma and 32 normal samples from TCGA. The clinical information is shown in Supplementary Table 1. The training set contained 15 normal samples and 147 cancer samples, and the test set contained 17 normal samples and 142 cancer samples. Univariate Cox analysis showed that T (HR = 2.347; P = 4.98e^-8^), N (HR = 2.814; P = 0.0004), M (HR = 1.048; P = 1.64e^-8^) (Supplementary Figure 1A) and 1547 protein-coding genes were prognostic factors. These risk ratios and significance of prognostic genes are shown in Supplementary Figure 1B.

### Robust prognostic gene selection

**Figure 2:**
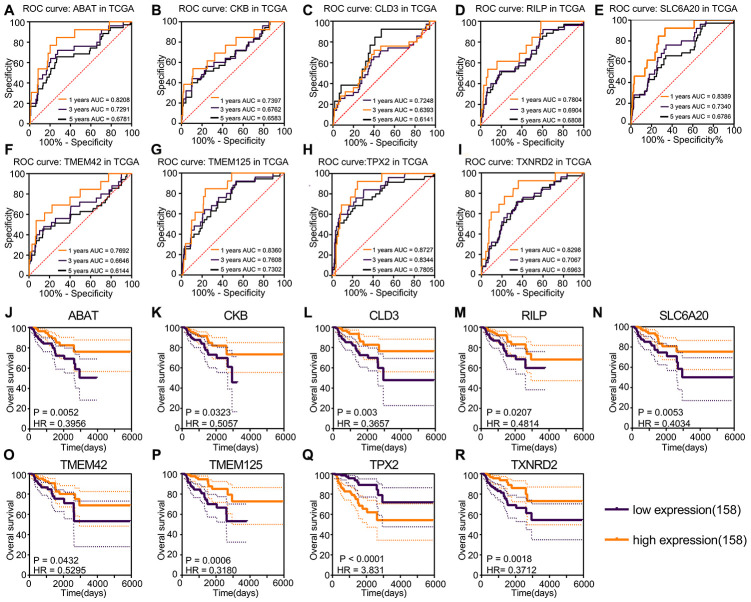
**Kaplan–Meier and ROC curves for prognosis factors generated using the “rbsurv” package.** (**A**–**I**) The ROC curve of risk factors and protective factors. *TPX2* acted as the only risk factor showed the largest AUC, 5-year AUC for *TPX2* was 0.7805. *ABAT, CKB, CLD3, RILP, SLC6A20, TMEM42, TMEM125* and *TXNRD2* acted as protective factors also showed accurate diagnosis ability. (**J**–**R**) Kaplan–Meier curves of risk factors and protective factors. The patients were divided into various risk groups according to expression levels. Nine prognostic factors showed significant survival difference and the largest HR was the 3.831 for *TPX2*, the lowest HR value was 0.3180 for THEM125.

A total of 1547 protein-coding genes were noise-reduced using false discovery rate (FDR); 297 prognosis genes were identified using FDR < 0.05. Nine prognosis genes were selected robustly using the “rbsurv” package based on these 297 prognosis genes. The univariate Cox regression P-value and FDR of the nine robust genes are shown in Table 1. The Akaike information criterion and negative log-likelihood of these nine genes are shown in Table 2. The survival analysis and ROC curves showed that *TPX2* was a risk factor, and that *ABAT, CKB, CLD3, RILP, SLC6A20, TMEM42, TMEM125, TXNRD2* were protective factors (Figure 2A–2R). *TPX2* was the only risk factor closely related to prognostic status (HR = 3.831; P < 0.0001) and had the ability to determine prognosis (1-year AUC = 0.8727; 3-year AUC = 0.7805; 5-year AUC = 0.7805).

**Table 1:** Univariate regression analyses of prognostic factors for overall survival.

**ID**	**P–valve**	**FDR**	**HR**	**95% CI of HR**
*TPX2*	2.97e–06	1.00e–03	2.093	1.535–2.853
*SLC6A20*	8.06e–06	2.00e–03	0.724	0.628–0.834
*RILP*	9.84e–06	2.00e–03	0.387	0.254–0.590
*CKB*	1.14e–05	2.00e–03	0.494	0.361–0.677
*TXNRD2*	1.53e–05	2.00e–03	0.301	0.175–0.519
*TMEM42*	2.92e–05	4.00e–03	0.450	0.310–0.654
*CLDN3*	3.62e–05	5.00e–03	0.716	0.610–0.839
*TMEM125*	6.44e–05	8.00e–03	0.690	0.576–0.828
*ABAT*	7.18e–04	3.50e–02	0.586	0.430–0.799

FDR: False discovery rate; HR: Hazard rate; CI: Confidence interval

**Table 2:** The prognosis–related model results selected by “rbsurv” package in R.

**Order**	**Gene**	**nloglik**	**AIC**	**Selected**
1	*CLDN3*	80.94	163.88	*
2	*TMEM125*	79.55	163.09	*
3	*TMEM42*	78.91	163.81	*
4	*CKB*	75.88	159.76	*
5	*TXNRD2*	74.48	158.95	*
6	*ABAT*	72.75	157.5	*
7	*RILP*	71.64	157.29	*
8	*SLC6A20*	69.42	154.84	*
9	*TPX2*	63.47	144.94	*
10	*DGLUCY*	62.97	145.94	

AIC: Akaike information criterion; nloglik: Negative log–likelihood

### Risk score model establishment and evaluation

**Figure 3:**
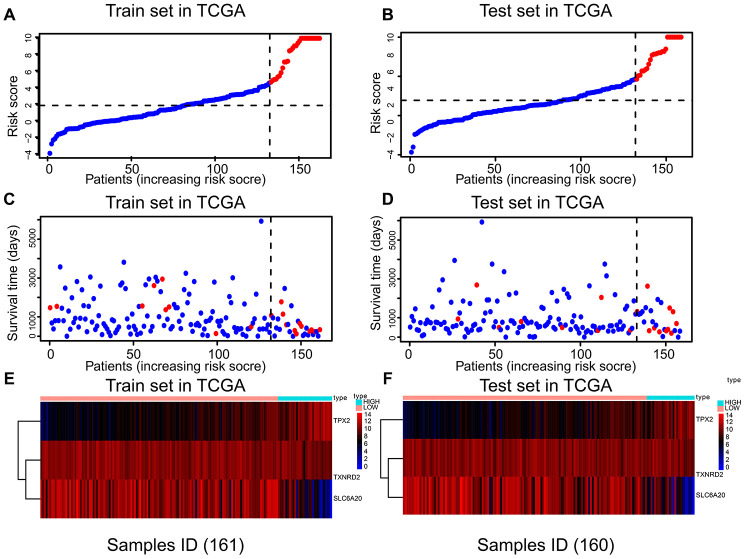
**The relation between risk score, survival state, and risk factors in the training and test sets.** There were three parts in training and test sets. The horizontal axis represents the same order of samples. (**A**, **B**) Parts A and B showing the risk score of each patient. (**C**, **D**) Parts C and D showing the survival state and risk score of each patient. (**E**, **F**) Parts E and F showing the expression levels of *TPX2*, *TXNRD2* and *SLC6A20*.

**Figure 4:**
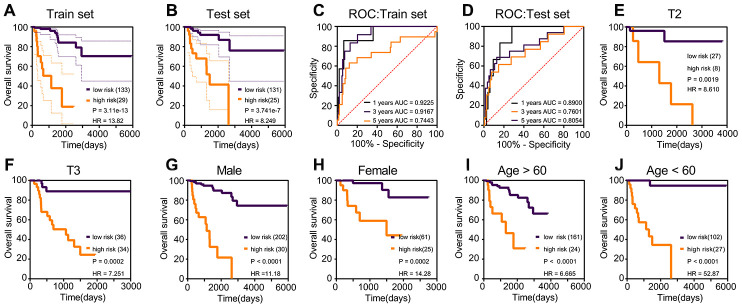
**Kaplan–Meier and ROC curves for risk score in the training set, test set and subgroups.** (**A**) The log-rank test p in training set was 3.11e^-13^, HR = 13.82. (**B**) The log-rank test p in test set was 3.741e^-7^, HR = 8.249. (**C**) The 1-year AUC of risk score in the training set was 0.9225; the 3-year AUC of risk score in the training set was 0.9167. (**D**) The 1-year AUC of risk score in the training set was 0.8900; the 3-year AUC of risk score in the training set was 0.7601. (**E**–**J**) The subgroups Kaplan–Meier analysis of risk score.

Risk scores were established using multivariate Cox regression (Table 3), based on the nine prognostic genes selected robustly. Risk score = 1.806 * *TPX2* - 0.355 * *TXNRD2* - 0.805 * *SLC6A20*. The optimal threshold score with the maximal sensitivity and specificity was 4.6846. The risk scores, survival statuses and gene expression levels corresponding to each sample are displayed in Figure 3A–3E. We found that patients in the high-risk score group had worse prognosis, and expression levels of *TPX2* were higher than those in the low-expression group. The risk score K–M analysis (P = 3.11e^-13^; HR = 13.82) and AUC (1-year = 0.9225; 3-year = 0.9167; 5-year = 0.7443) in the training set were consistent with the results of risk score on K–M analysis (P = 3.741e^-7^; HR = 8.249) and AUC (1-year = 0.890; 3-year = 0.7601; 5-year = 0.8054) in the test set (Figure 4A–4D). Subsequently, we evaluated the prognostic ability of risk score in the various subgroups of TCGA, and found that the risk score had important prognostic value in the subgroups such as T2, T3, gender, and age (Figure 4E–4H).

**Table 3:** Multivariate Cox regression analyses of prognostic factors for overall survival.

**ID**	**P–valve**	**HR**	**95% CI of HR**
*TPX2*	0.001	1.806	1.287–2.533
*TXNRD2*	0.001	0.355	0.192–0.658
*SLC6A20*	0.007	0.805	0.689–0.941

HR: Hazard rate; CI; Confidence interval

### Weighted gene co-expression network

**Figure 5:**
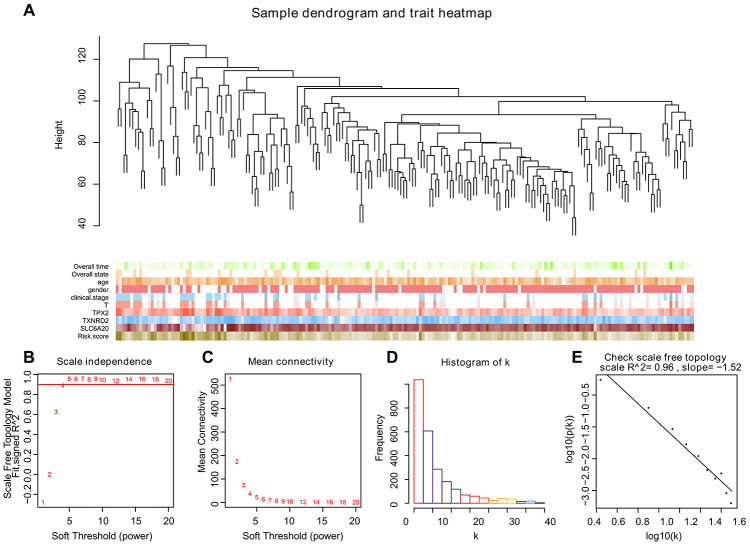
**Selecting the appropriate beta value to build the cluster tree.** (**A**) Sample tree and phenotype relation heat map, we selected the cutoff point of 130 and obtained 198 clinical samples. The phenotype includes overall survival, overall state, age, gender, clinical stage, and the expression level of *TPX2*, *TXNRD2* and *SLC6A20*. The larger the value, the darker the color. (**B**–**E**) We built scale-free co-expression networks. The best soft threshold was 5, R-squared = 0.96.

**Figure 6:**
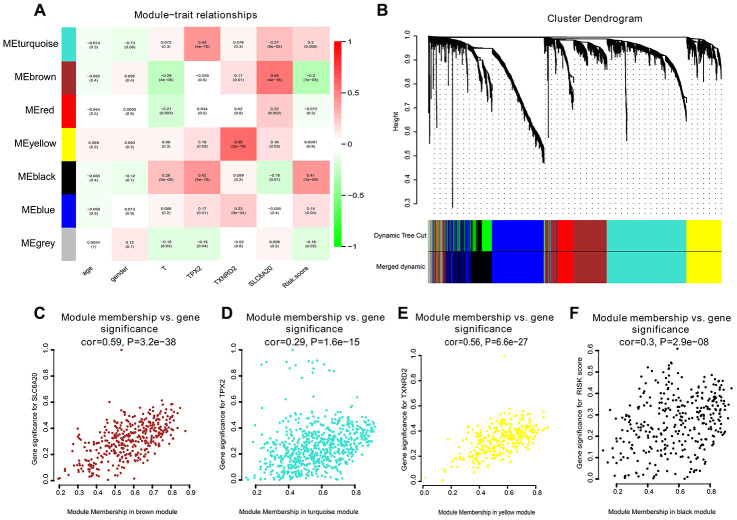
**Correlation heat map among various phenotype and co-expression modules.** (**A**) The correlation heat map. (**B**) The hierarchical clustering tree was showed, where each leaf on the tree represents a gene, and each branch represents a co-expression module. (**C**) *SLC6A20* co-expression module factors were shown. The horizontal axis is module membership; the vertical axis is gene significance. (**D**) *TPX2* co-expression module factors. The horizontal axis is module membership; the vertical axis is gene significance. (**E**) *TXNRD2* co-expression module factors were shown. The horizontal axis is module membership; the vertical axis is gene significance. (**F**) Risk score correlation module. The horizontal axis is module membership; the vertical axis is gene significance.

To create co-expression networks of *TPX2*, *TXNRD2*, SLC206A and risk score, we selected the cutoff point of 130 and obtained 198 clinical samples. A sample cluster map and phenotype-related heat map were constructed (Figure 5A). We set the soft threshold as 5 (Figure 5B, 5C), R square = 0.96 (Figure 5D, 5E), and established a scale-free network. We set the number of genes in the minimum module as 30, abline as 0.25. We drew correlation heat maps for modules and genes (Figure 6A). The hierarchical clustering tree is shown in Figure 6B, where each leaf on the tree represents a gene, and each branch represents a co-expression module. We found that the brown module has the strongest positive correlation with *SLC6A20* (Pearson Cor = 0.59; P = 3.2e^-8^) (Figure 6C). The turquoise module had the strongest positive correlation with *TPX2* (Pearson Cor = 0.29; P = 1.6e^-15^) (Figure 6D), the yellow module had the strongest positive correlation with *TXNRD2* (Pearson Cor = 0.56; P = 6.6e-27) (Figure 6E) and the black module had the strongest positive correlation with risk score (Pearson Cor = 0.30; P = 2.9e-8) (Figure 6F). The correlation between co-expression factors and risk score are shown in Supplementary Figure 2.

### PPI, function analysis and GSEA

**Figure 7:**
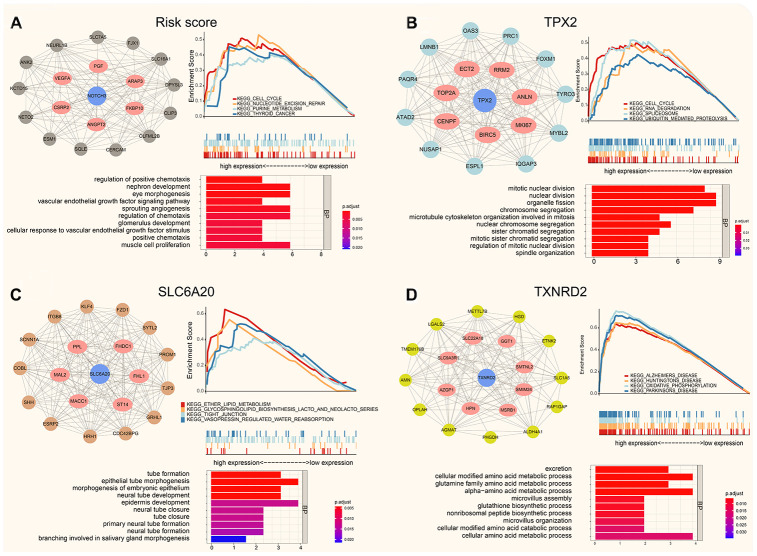
**The protein-protein interaction network, function enrichment and GSEA analysis of co-expression modules (Pearson Cor > 0.4).** (**A**) The risk score correlation genes in black module. (**B**) The co-expressed genes for *TPX2* in turquoise module. (**C**) The co-expression genes for *SLC6A20* in brown module. (**D**) The *SLC6A20* co-expression genes in yellow module.

Using WGCNA, we obtained the co-expression modules of *TPX2*, *TXNRD2*, *SLC6A20* and risk score, the PPI (protein-protein interaction network), Function analysis and GSEA of them are shown in Figure 7. GO analysis showed that the top 20 co-expression genes of risk score were significantly enriched in regulation of positive chemotaxis, nephron development, and sprouting angiogenesis. GSEA analysis of risk score showed that cell cycle, nucleotide excision repair and purine metabolism were related to the high expression group (Figure 7A). GO analysis showed that the top 20 co-expression genes of *TPX2* were significantly enriched in mitotic nuclear division, nuclear division, chromosome segregation. GSEA analysis of *TPX2* showed that cell cycle, RNA degradation and spliceosome were related to the high expression group of *TPX2* (Figure 7B). GO analysis showed that the top 20 co-expression genes of *SLC6A20* were significantly enriched in tube formation, epithelial tube morphogenesis, and neural tube development. GSEA analysis of *SLC6A20* showed that ether lipid metabolism, tight junction, and vasopressin regulated water reabsorption were related to the high expression group of *SLC6A20* (Figure 7C). GO analysis showed that the top 20 co-expression genes of *TXNRD2* were significantly enriched in excretion, cellular modified amino acid metabolic process, and glutamine family amino acid metabolic process. GSEA analysis of *TXNRD2* showed that Alzheimer disease, Huntington disease, Parkinson disease were related to the high expression group of *TXNRD2* (Figure 7D).

### Relationship between immune infiltration and risk score

**Figure 8:**
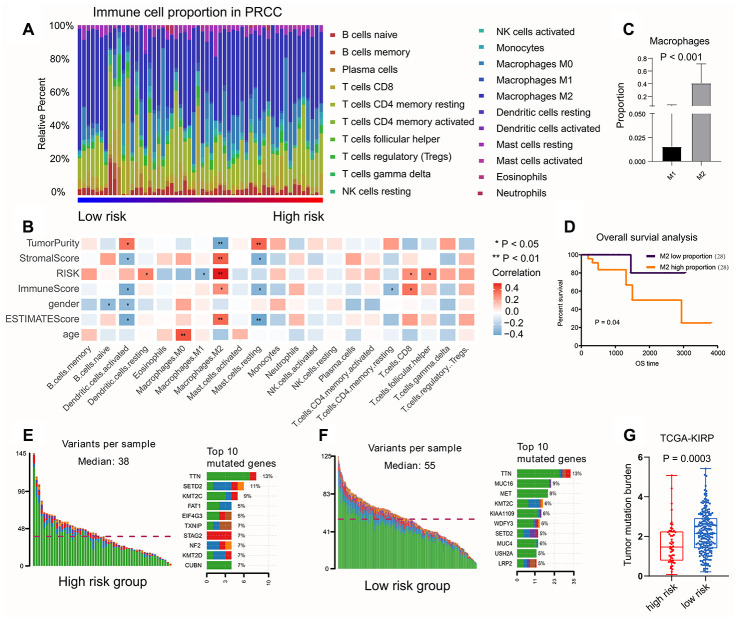
**The relationship between risk score and immune infiltration, TMB value.** (**A**) The percentage of immune cell content in the various samples was showed, M2 macrophages accounted for the highest proportion. (**B**) The correlation between the proportion of immune cells in the samples and the risk score. M2 macrophages were positively related to risk score, while the M1 macrophages were negatively related to risk score. (**C**, **D**) The survival analysis to show M2-type macrophages account more proportion and lead a worse prognosis compared with M1-type macrophages. (**E**, **F**) Somatic mutations of *TTN* (P < 0.001) was higher both in low-risk group and high-risk group. (**G**) The patients with low risk score had higher TMB value, suggesting that the patients experienced better effects on the immune response and had a better prognosis.

We calculated the percentage of immune cell content in the various samples using the CIBERSORT method with P < 0.05, and obtained 56 tumor samples. We found that M2 macrophages accounted for the highest proportion (Figure 8A). We also calculated the correlation between the proportion of immune cells in the samples and the risk score (Figure 8B). We found that the M2 macrophages were positively related to risk score, while the M1 macrophages were negatively related to risk score. Macrophage polarization plays an important role in tumor progression; M1 macrophages play a protective role, while M2 macrophages promote tumor growth. Therefore, we performed survival analyses; these showed that M2 macrophages accounted for higher proportions and led to worse prognosis compared with M1 macrophages (Figure 8C, 8D).

### Relationship between TMB value and risk score

We first evaluated the variation in each TCGA-KIRP sample. the results showed that somatic mutations of *TTN* (P < 0.001) were greater in number both in low-risk group and high-risk group (Figure 8E, 8F). Patients with low risk scores had higher TMB value, suggesting that patients had better results in terms of immune responses and had better outcomes (Figure 8G).

### Pan-cancer analysis

**Figure 9:**
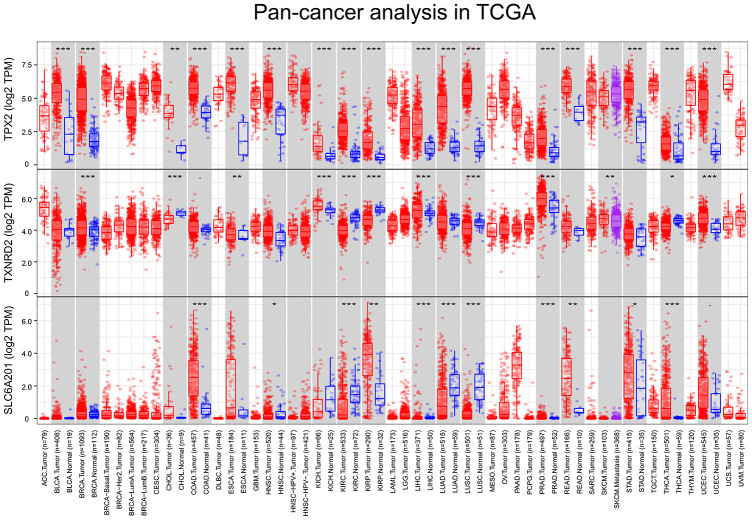
The variation analysis of 33 types of cancer in TCGA, P <0.05 was marked as *, P < 0.01 was marked as **, P < 0.001 was marked as ***. (**A**) TPX2, (**B**) TXNRD2, (**C**) SLC6A20.

**Figure 10:**
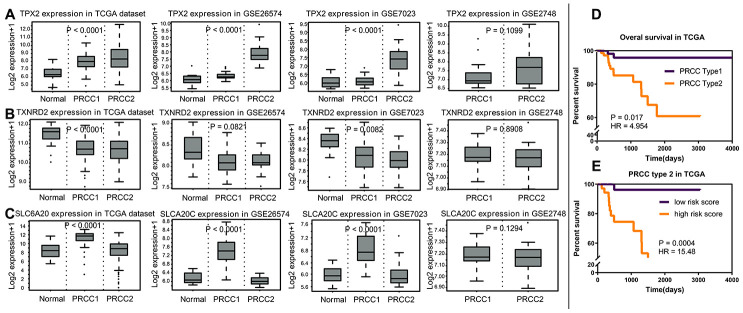
**The analysis of variance of normal renal tissue and papillary renal carcinoma.** (**A**) In TCGA, GSE26574, GSE7023 and GSEA2748, *TPX2* acted as prognostic risk factors, upregulated significantly in PRCC2. (**B**) *TXNRD2* acted as prognostic protective factor, downregulated significantly in the PRCC group. (**C**) *SLC6A20* acted as prognostic protective factor, downregulated significantly in the PRCC2 group, compared with PRCC1. (**D**) PRCC2 showed worse prognosis compared with PRCC1 (P = 0.017; HR = 4.954). (**E**) Based on the cut off as 4.6846, the PRCC type 2 group patients were divided into two risk groups, and the high-risk group showed the worse prognosis status (P = 0.0004; HR = 15.48).

The expression differences of *TPX2*, *TXNRD2* and *SLC6A20* in 33 cancers in TCGA are shown in Figure 9. *TPX2* was upregulated in kidney chromophobe (P < 0.001), kidney renal clear cell carcinoma (P < 0.001), and kidney renal papillary cell carcinoma compared to normal tissue. *TXNRD2* was downregulated in kidney chromophobe (P < 0.001), kidney renal clear cell carcinoma (P < 0.001), and kidney renal papillary cell carcinoma compared to normal tissue. The results of *TPX2* and *TXNRD2* differential expression analysis were consistent with their effect on patient prognosis. However, *SLC6A20* was downregulated in kidney chromophobe (P < 0.001), kidney renal clear cell carcinoma (P < 0.001), but upregulated in kidney renal papillary cell carcinoma compared to normal tissue. *SLC6A20* acted as a protective prognostic factor and should be downregulated in the tumor group; however, in TCGA, the result was the opposite. To explain this result, we analyzed the differential expression of *TPX2*, *TXNRD2* and *SLC6A20* in PRCC type 1 and PRCC type 2. We found that *TPX2* was upregulated significantly in the PRCC type 2 group (Figure 10A), *TXNRD2* and *SLC6A20* downregulated significantly in the PRCC type 2 group (Figure 10B, 10C). PRCC type 2 gave worse outcomes than did PRCC type 1 (Figure 10D, 10E). These findings suggest that *SLC6A20* is downregulated significantly in PRCC2 compared to PRCC1, and that *SLC6A20* acts as a prognostic protective factor.

### Knockdown of *TPX2* significantly inhibited cell proliferation and invasion

**Figure 11:**
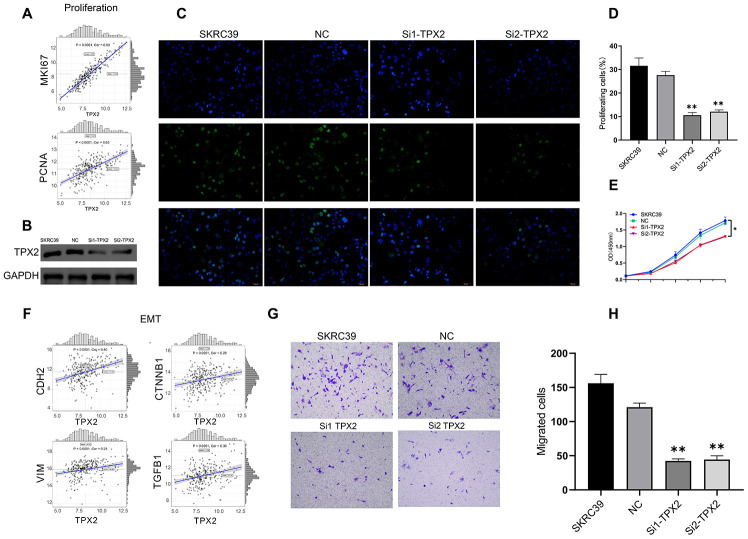
**Experiment of *TPX2*.** (**A**) The correlation analysis between *MKI67*, *PCNA* and *TPX2*. (**B**) Immunoblot analysis of *TPX2* protein in SKRC39 cells following *TPX2* knockdown. GAPDH served as loading control. (**C**, **D**) EdU incorporation assays were used to determine the effects of *TPX2* on SKRC39 cell proliferation. The ratio of EdU-positive cells (green) per field to the number of Hoechst 33342-positive cells (blue) in the same field was calculated in five random fields. (**E**) The effects of *TPX2* on the proliferation of SKRC39 cells were analyzed by CCK-8 assays. The results are presented as the mean optical density (OD) at 450 nm for triplicate wells. The results are presented as the mean ± SD of three independent experiments (*P < 0.05, **P < 0.01). (**F**) The correlation analysis between *CDH2*, *CTNNB1*, *VIM*, *TGFB1* and *TPX2*. (**G**, **H**) Knockdown of *TPX2* inhibited cell migration as detected by Transwell assays. Number of cells that invaded through the membrane was counted in 10 fields under magnification, ×200; compared with NC and SKRC39 control group.

*TPX2* is overexpressed in papillary renal cell carcinoma and is related to poor prognosis. In PRCC cell line, the role of *TPX2* was determined *in vitro* experiments. First of all, *TPX2* showed positively correlation to *MKI67* and *PCNA* in TCGA (Figure 11A). Small interfering RNAs was used to reduce expression levels of *TPX2* in the papillary renal cell carcinoma cell line SKRC39 (Figure 11A) and the *TPX2* protein expression level were shown in Figure 11B. EdU incorporation assays were used to determine the effects of TPX2 on SKRC39 cell proliferation. EdU assay suggested that *TPX2* inhibits PRCC cell lines proliferation capacity (Figure 11C and 11D). The effects of *TPX2* on the proliferation of SKRC39 cells were analyzed by CCK-8 assays. The results are presented as the mean ± SD of three independent experiments (*P < 0.05, **P < 0.01) (Figure 11E). Afterwards, *TPX2* showed positively correlation to *CDH2*, *CTNNB1*, *VIM* and *TGFB1* in TCGA (Figure 11F). The results of transwell assays showed that Knockdown of *TPX2* inhibited cell migration (Figure 11G and 11H).

## DISCUSSION

Papillary renal cell carcinoma carries a poor prognosis. In this paper, a prognostic score model for patients with papillary renal cell tumors was constructed. Our prognostic score distinguished patients with poor prognosis in T2 and performed determined prognosis in various subgroups. The prognostic score of papillary renal cell carcinoma was established based on TCGA and was verified using the test set. The scoring model contained three factors *TPX2*, *TXNRD2*, and *SLC6A20*. *TPX2* was a risk factor and *TXNRD2* and *SLC6A20* were protection factors. We analyzed their biological functions and related regulation pathways. Furthermore, M2-type macrophages and tumor mutation burden showed close correlation to risk, illustrating the important biological significance of the risk score.

There have been studies to predict the risk model of papillary renal cell carcinoma, and the prediction effect was adequate; however, there were too many factors in the model, and the predictive abilities of single factors were not strong, suggesting that they cannot be used as optimal prognostic biomarkers. In our studies, we conducted a risk score composed by only three genes, and the predictive abilities of single factors was strong.

There are three factors in our risk signature. The microtubule-associated protein *TPX2* [10, 11] was a risk factor in the risk score. We identified its prognostic co- expression module biological process, and found that *TPX2* co-expressed genes were significantly enriched in cell cycle process, mitotic cell, and cell division. The division and proliferation of cells depend on a normal cell cycle, and unscheduled proliferation is one of the hallmarks of tumors [12].

Cell cycle regulators are also directions for promising tumor treatments [13]. DNA topoisomerase 2a (*TOP2A*) and centromere protein F (*CENPF*) were the strongest co-expression genes of *TPX2*, and have been studied in many tumors. *TOP2A* plays an important role in promoting tumorigenesis in breast cancer [14], ovarian cancer [15], prostate cancer [16], colon cancer [17], and acts as biomarker in renal clear cell cancer prognosis status [18]. *CENPF* has also been reported as a prognostic factor for breast cancer [19], renal cancer [20], and bladder cancer [21]. Therefore, we believe that factors in the *TPX2* co-expression module might affect the prognosis of papillary renal cell carcinoma by participating in the process of regulating cell proliferation, and we verified it in function experiment.

In the *TPX2* co-expression module, the synergistic effect between *CENPF* and *FOXM1* were found to activate prostate malignancies; experimental verification showed that the synergistic effect of *FOXM1* and *CENPF* promoted tumor growth by activating key signaling pathways and acted as an important marker of tumor prognosis and survival [22]. Using GSEA analysis of the *TPX2* prognostic co-expression module, we found that co-expressed *TPX2* genes were closely related to cell cycle, RNA degradation and spliceosome. These findings suggested that the co-expression module of *TPX2* had important biological significance.

*TXNRD2* and *SLC6A20* are protective factors for PRCC. Thioredoxin reductase 2 (*TXNRD2*), the protein encoded by this gene belongs to the pyridine nucleotide-disulfide oxidoreductase family, and is a member of the thioredoxin (Trx) system. Wang et al. suggested *TXNRD2* polymorphisms acted as the protective factors of gastric cancer [23]. Solute carrier family 6 member 20 (*SLC6A20*), the protein encoded by this gene is a member of the subgroup of transporter with unidentified substrates within the Na+ and Cl- coupled transporter family, expressed in kidney [24]. To the best of our knowledge, this is the first study to identify *TXNRD2* and *SLC6A20* as PRCC prognosis factors. Nevertheless, there are few studies of the direction of papillary renal cell carcinoma and further research is needed to explore this area.

Papillary renal cell carcinoma is characterized by various immune microenvironments. Immune infiltration analysis showed that the risk score had the strongest correlation with M2 macrophages proportion. Tumor associated macrophages (TAMs) play important roles in tumor microenvironments. In renal cell carcinoma, a higher density of M2 macrophages was associated with worse prognosis, while M1 macrophages had the opposite effect [25]. Our present findings are consistent with these results, and illustrate the role of immune infiltration in papillary renal cell carcinoma.

We also explored the role of tumor mutation burden in PRCC prognostic status. Tumor mutation burden is considered a biomarker for immunotherapy. It is currently believed that tumors with high mutation burden are more likely to be detected and killed by the immune system, and to generate a stronger immune response [26]. In our studies, we found the samples in high risk group had higher TMB value. Nevertheless, the ability of TMB to predict immunotherapy in PRCC needs further study.

In conclusion, we established an accurate prognostic prediction model, and built papillary renal cell carcinoma prognostic co-expression networks. We identified biological process and related pathways of these prognostic modules. M2 macrophages and TMB were related to risk score. Nevertheless, the specific upstream and downstream regulation and mechanisms require further exploration.

## MATERIALS AND METHODS

### Data acquisition and processing

The expression matrix and clinical information of renal papillary cell carcinoma were obtained from TCGA (http://cancergenome.nih.gov/). The expression matrix was normalized by log_2_ (exp + 1). Among protein-coding genes, genes with median and variance of the top 75% were used as the input data for this paper (Supplementary Table 2). The data set was randomly grouped based on random number method using SPSS software 22.0 (Supplementary Table 3). We obtained the expression matrix and platform comment information of GSE26574 [27], GSE7023 [28], GSE 2748 [29] from the GEO Gene Expression Omnibus (GEO) (https://www.ncbi.nlm.nih.gov/geo/).

### Prognostic factors selection

The “Survival” package [30] was used to obtain prognostic factors. The screening principle was P < 0.05. The false discovery rate was used to reduce noises and false positives with FDR < 0.05. The corresponding hazard ratio and P-value were displayed using GraphPad Prism 8.

### Identification robust prognostic genes

Subsequently to obtain robust survival associated genes, we performed robust likelihood-based survival analyses based on the prognostic genes selected by univariable Cox regression analysis (FDR<0.05). Robust prognosis-related genes were selected using the “Rbsurv” package [31–33], iteration = 100, max concern gene = 30. Kaplan–Meier [34] and ROC curves [35] were used to evaluate the prognostic value of these genes, and the log-rank [36] test was used to calculate the significance. The algorithm of *rbsurv* package was showed in Supplementary Table 4.

### Establishment of risk scores

Multivariate Cox regression analysis was performed to obtain a prognostic correlation model among the nine robust prognostic factors using SPSS 22.0 software (Supplementary Table 5). In the model, the hazard ratio was used as the coefficient: Risk factors hazard ratio > 1, protection factors hazard ratio < 1. We calculated the risk score for each patient, ranked the patients based on the risk score, and used the “pheatmap” package to determine the expression levels of risk factors. Finally, the risk score was evaluated in the training set, the test set and the various subgroups.

### Weighted gene co-expression network and GSEA

The “WGCNA” [37] R package was used to construct co-expression modules for risk factors. Then we selected the top co-expression genes with risk factors (Pearson Cor > 0.4). We constructed protein-protein interaction networks and presented it using Cytoscape. To clarify the relevant functions of risk factors, DAVID [38] was used to perform functional enrichment analysis and screen biological processes among the top co-expression genes. Gene set enrichment analysis (GSEA) is a calculation method that determines the significance and consistency differences of a predefined dataset between two biological states [39]. The gene matrix in TCGA was divided into high and low expression groups, in accordance with the median expression level of prognostic genes in model. Based on allocation, biological functions related to the high expression group was identified, allowing us to identify the mechanisms underlying the role of prognostic genes in model.

### Relationship between immune infiltration and risk score

The Estimation of Stromal and Immune cells in Malignant Tumor tissues using Expression data (ESTIMATE) is a method that infers the fraction of stromal and immune cells using gene expression signatures [40]. Using the ESTIMATE algorithm, we calculated stromal scores, immune scores, and tumor purity in each papillary renal cell carcinoma sample. CIBERSORT is an algorithm that analyzes the cell proportion in bulk tissue gene expression matrices. LM22 is a gene signature matrix that defines 22 immune cell subtypes [41]. This was download from the CIBERSORT website portal (https://cibersort.stanford.edu/). We analyzed immune cell proportions based on the LM22 matrix and CIBERSORT algorithm, and samples with P < 0.05 were considered to be significant and were considered in this study. A total of 56 samples were selected and are shown in Supplementary Table 6. Based on the robust risk score, we divided the PRCC samples into high and low risk groups, and analyzed the immune cell proportion difference in different risk groups. The correlations between risk score and immune cell infiltration were calculated.

### Relationship between TMB value and risk score

TMB is a measure of the total number of mutations per megabyte in a chromosome. This includes the total number of base substitution inserts, gene coding errors and deletions [42]; 38 MB is usually based on the length of human exons, and TMB is estimated to be equal to the total mutation frequency /38 Mb. TMB per megabyte is calculated by dividing the total number of mutations by the size of the target coding region.

### Pan-cancer analysis of prognostic genes

The Tumor Immune Estimation Resource (TIMER; https://cistrome.shinyapps.io/timer/) [43] was used to analyze the difference expression of *TPX2*, *TXNRD2* and *SLC6A20* between normal tissue and cancer in 33 cancer types.

### Cell culture and siRNA-PTEN construction

The SKRC39 cells were cultivated in DMEM(HighGlucose) with 10% of fetal calf serum, 100 U/ml penicillin and 100 μg/ml streptomycin. The cells were cultured at 37 °C, with 5% CO2. The *TPX2* (H) - 951 siRNA sequences were as follows: 5’-CCUGUAAUCAUCGAUGAAATT-3’ 5’-UUUCAUCGAUGAUUACAGGTT-3’. The *TPX2* (H) - 2046 siRNA sequences were: 5’-GCUCAACCUGUGCCACAUUTT-3’ 5’-AAUGUGGCACAGGUUGAGCTT-3’.

### Western blotting (WB)

TPX2 protein content was taken from the transfected SKRC39 cell total proteins and were lysed in radioimmunoprecipitation assay (RIPA) buffer. After 15min centrifugation, the supernatant was collected and injected with the bicinchoninic acid assay kit to detected protein concentrations. 10% SDS-PAGE was used to separate the proteins, and the proteins were transferred to polyvinylidene fluoride membranes. Afterwards, we added 5% non-fat milk and sealed the membranes at 37 °C for 1 hour. After the addition of primary antibodies specific for TPX2 at 4 °C. Blots were washed with TBST for 3 times to staining for 2 h at room temperature with secondary antibodies. An EasySee Western Blot kit (Beijing Transgen Biotech, Beijing, China) was then used for protein visualization, with ImageJ software being used to measure protein expression.

### Cell proliferation assay

SKRC39 treated cells were seeded in 96-well plates, Cell Counting Kit-8 (CCK-8) assay reagent (Dojindo Molecular Technologies, Kumamoto, Japan) was added according to the manufacturer’s instructions, and the OD values were measured by an absorbance reader (Bio-Rad) at wavelength 450 nm.

### Transwell assay

Transwell chambers with 8-μm pores matched with 24-well plates were used for cell migration assays. 600 μl of medium (10% FBS) was added to the plate bottom of the chamber. Then, a certain number of SKRC39 cells were re-suspended with 200 ul serum-free medium and added to the upper chamber. After incubation at 37°C for 36 h, total cells remained on the upper chamber were removed using cotton swabs, and those that had migrated to the lower side were fixed with 4% paraformaldehyde for 10 min, and stained with 1.0% crystal violet for 10 min at room temperature. After the chambers were washed by 1 × PBS, the images were captured by EVOSTM XL Core Imaging system (Invitrogen; Thermo Fisher Scientific, Inc.) and dealt with ImageJ software.

### Ethynyl-20-deoxyuridine assay

The 5-ethynyl-20-deoxyuridine (EdU) assay kit (Ribobio, Guangzhou, China) was utilized to measure cell proliferation. Briefly, cells were seeded into each well of 24-well plates for 24 h and then cultured in the medium contained 50 μM EdU for 2 h. Subsequently, the cells were then fixed with 4% paraformaldehyde, incubated for 30 minutes with 100 μl Click Additive Solution and stained with 100 μl DAPI. Fluorescence microscope (Olympus, BX51 TRF, USA) was used to visualize EdU positive cells and the Image J software (NIH Image, Bethesda, MD, USA) was used to count the percentage of EdU-positive cells.

## Supplementary Material

Supplementary Figures

Supplementary Table 1

Supplementary Table 2

Supplementary Table 3

Supplementary Table 4

Supplementary Table 5

Supplementary Table 6
