# Supplementary material for: Three-gene risk model in papillary renal cell carcinoma: a robust likelihood-based survival analysis
Source: Aging (Albany NY). 2020 Nov 5;12(21):21854–73. doi: 10.18632/aging.104001 (PMC7695399; doi:10.18632/aging.104001)
Supplement: Supplementary Figures [file aging-12-104001-s001..pdf]

SUPPLEMENTARY FIGURES

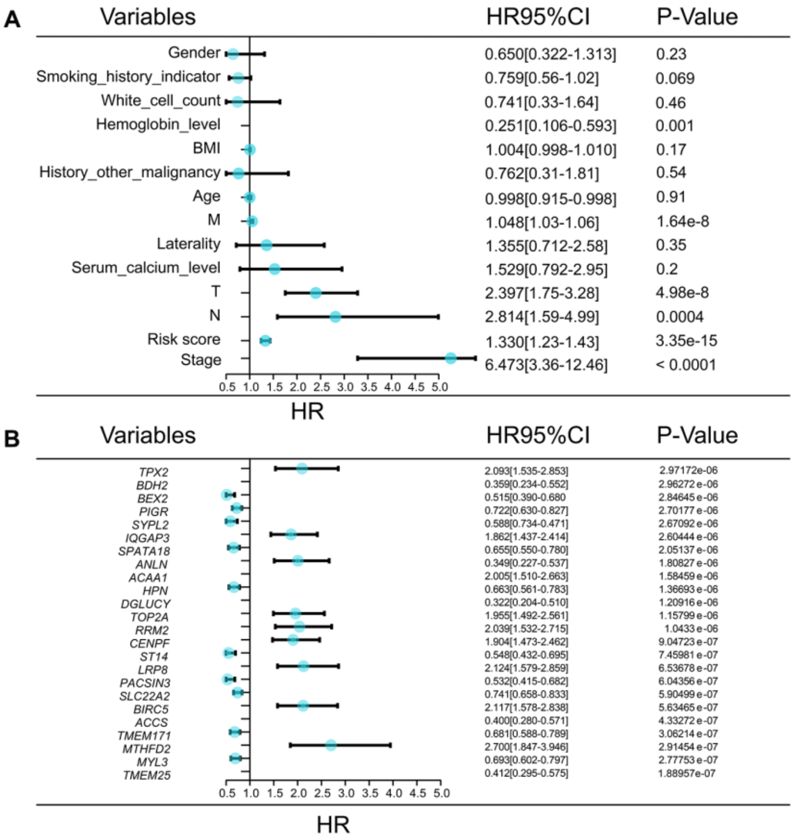

Supplementary Figure 1. Forest plot of prognostic factors. (**A**) The clinical prognostic factors are shown, including HR and P-values. (**B**) The protein coding prognostic factors are shown, including HR and P-values.

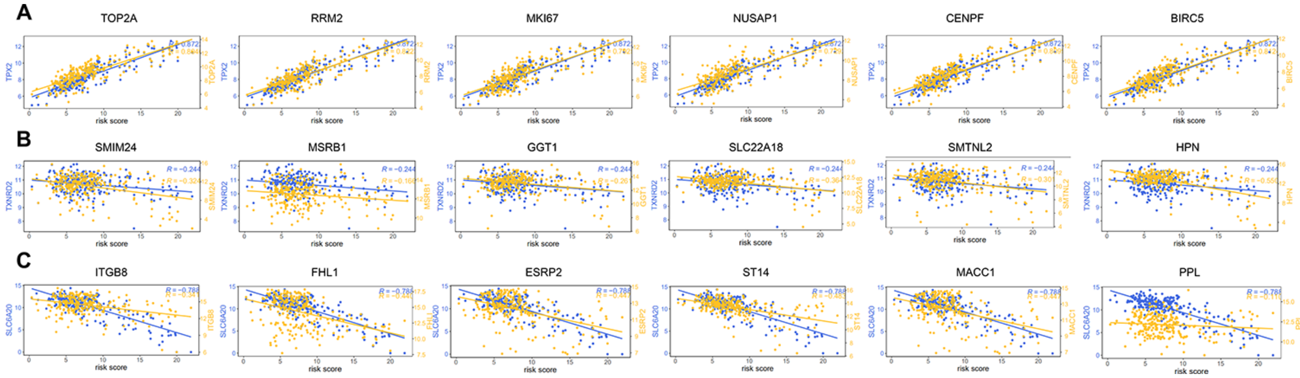

Supplementary Figure 2. The correlation between risk score and the co-expression genes of (**A**) TPX2, (**B**) *TXNRD2* and (**C**) *SLC6A20*.
