# Supplementary material for: Three-gene risk model in papillary renal cell carcinoma: a robust likelihood-based survival analysis
Source: Aging (Albany NY). 2020 Nov 5;12(21):21854–73. doi: 10.18632/aging.104001 (PMC7695399; doi:10.18632/aging.104001)
Supplement: Supplementary Table 1 [file aging-12-104001-s002..pdf]

SUPPLEMENTARY TABLE

Supplementary Table 1. Baseline of renal papillary cancer in TCGA.

Type		
Normal	32	10.00%
Tumor	288	90.00%
**Status**		
Alive	248	86.10%
Dead	40	13.90%
**Age**		
≥ 60	169	58.70%
<*60*	116	40.30%
NA	3	1.00%
**Gender**		
Male	212	73.60%
Female	76	26.40%
**Clinical_stage**		
I	172	59.70%
II	21	7.30%
III	51	17.70%
IV	15	5.20%
NA	29	10.10%
Clinical_M		
M0	95	33.00%
M1	9	3.10%
MX	170	59.00%
NA	14	4.90%
**Clinical_N**		
N0	49	17.00%
N1	24	8.30%
N2	4	1.40%
NX	210	73.00%
NA	1	0.30%
**History_other_malignancy**		
No	233	80.90%
Yes	55	19.10%
**Body Mass Index**		
Normal (*18.5-24.9*)	50	17.40%
Overweight (*25-29.9)*	86	29.90%
Mild obesity (*30-34.9*)	41	14.20%
Moderate obesity (≥*35*)	35	12.10%
NA	76	26.40%
**AJCC-T**		
T1	193	68.06%
T2	32	11.11%
T3	59	20.49%
T4	2	0.69%
NA	2	0.69%
