# Supplementary material for: Three-gene risk model in papillary renal cell carcinoma: a robust likelihood-based survival analysis
Source: Aging (Albany NY). 2020 Nov 5;12(21):21854–73. doi: 10.18632/aging.104001 (PMC7695399; doi:10.18632/aging.104001)
Supplement: Supplementary Table 4 [file aging-12-104001-s005..pdf]

SUPPLEMENTARY TABLE

Supplementary Table 4. The algorithm of rbsurv package was showed.

1. We randomly divided the training set (N = 147 samples) into the sub-training set with N*(1-p) samples and the sub-validation set with N * p samples, p = 1/3. We fit a gene to the sub-training set of samples and obtain the parameter estimate. Then evaluate log likelihood with the parameter estimate and the sub-validation set. Perform this evaluation for each gene.
2. We repeat the procedure above 100 times, thereby obtaining 100 times log likelihood for each gene. We then selected the best gene with the smallest mean negative log likelihood (or the largest mean log likelihood). The best gene is the most survival-associated one that is selected by the robust likelihood-based approach.
3. We let gene A be the selected best gene in the previous step. We found the next best gene B by repeating the previous two steps.
4. We continued this selection until there was a lack of samples, resulting in a series of K models M1 = A, M2 = A+B, MK = A+B+…+K.
5. We selected the best genes in the model with the smallest AIC.
