# Supplementary material for: Three-gene risk model in papillary renal cell carcinoma: a robust likelihood-based survival analysis
Source: Aging (Albany NY). 2020 Nov 5;12(21):21854–73. doi: 10.18632/aging.104001 (PMC7695399; doi:10.18632/aging.104001)
Supplement: Supplementary Table 5 [file aging-12-104001-s006..docx]

**Supplementary Table 5. The process of Multivariate Cox regression analysis.**

| **Omnibus Tests of Model Coefficients** | | | | | | | | | | |
| --- | --- | --- | --- | --- | --- | --- | --- | --- | --- | --- |
| Step | -2 log likelihood | Overall (score) | | | Change From Previous Step | | | Change From Previous Step | | |
|  |  | Chi-square | Df | Sig | Chi-square | Df | Sig | Chi-square | Df | Sig |
| 1^a^ | 151.935 | 24.901 | 1 | 6.03E^-7^ | 22.475 | 1 | 2*10^-6^ | 22.475 | 1 | 2*10^-6^ |
| 2^b^ | 139.771 | 40.404 | 2 | 1.68E^-9^ | 12.165 | 1 | 4*10^-4^ | 34.640 | 2 | 3.00E^-8^ |
| 3^c^ | 132.938 | 71.373 | 3 | 2.16E^-15^ | 6.833 | 1 | .009 | 41.473 | 3 | 5.19E^-9^ |
| a. Variable(s) Entered at Step Number 1 :TPX2 | | | | | | | | | | |
| b. Variable(s) Entered at Step Number 2 :TXNRD2 | | | | | | | | | | |
| c. Variable(s) Entered at Step Number 3 :SLC6A20 | | | | | | | | | | |
| d. Beginning Block Number 1. Method = Forward Stepwise (Likelihood Ratio) | | | | | | | | | | |
| Df: Degree of freedom; Sig: Significance. | | | | | | | | | | |

| **Variables in the equation** | | | | | | | |
| --- | --- | --- | --- | --- | --- | --- | --- |
|  | | B | SE | Wald | Df | Sig | Exp(B) |
| Step 1 | TPX2 | .739 | .158 | 21.835 | 1 | 3*10^-6^ | 2.093 |
| Step 2 | TPX2 | .717 | .164 | 19.085 | 1 | 1.3*10^-5^ | 2.048 |
|  | TXNRD2 | -1.071 | .282 | 14.473 | 1 | 1.4*10^-4^ | .343 |
| Step 3 | TPX2 | .591 | .173 | 11.711 | 1 | .001 | 1.806 |
|  | TXNRD2 | -1.035 | .314 | 10.845 | 1 | .001 | .355 |
|  | SLC6A20 | -.217 | .080 | 7.373 | 1 | .007 | .805 |
| Df: Degree of freedom; Sig: Significance. | | | | | | | |

| **Variables not included in the equation^a,b,c^** | | | | |
| --- | --- | --- | --- | --- |
|  | | Score | Df | Sig |
| Step 1 | TMEM42 | 9.751 | 1 | .002 |
|  | RILP | 9.396 | 1 | .002 |
|  | TMEM125 | 5.276 | 1 | .022 |
|  | TXNRD2 | 15.062 | 1 | .001 |
|  | SLC6A20 | 11.850 | 1 | .001 |
|  | ABAT | 12.723 | 1 | .001 |
|  | CLDN3 | 9.944 | 1 | .002 |
|  | CKB | 6.917 | 1 | .009 |
| Step 2 | TMEM42 | 2.006 | 1 | .157 |
|  | RILP | 1.392 | 1 | .238 |
|  | TMEM125 | 2.118 | 1 | .146 |
|  | SLC6A20 | 8.064 | 1 | .005 |
|  | ABAT | 7.332 | 1 | .007 |
|  | CLDN3 | 2.962 | 1 | .085 |
|  | CKB | .939 | 1 | .332 |
| Step 3 | TMEM42 | .312 | 1 | .577 |
|  | RILP | .800 | 1 | .371 |
|  | TMEM125 | .002 | 1 | .967 |
|  | ABAT | 1.842 | 1 | .175 |
|  | CLDN3 | .124 | 1 | .725 |
|  | CKB | .371 | 1 | .542 |
