# Supplementary material for: Three-gene risk model in papillary renal cell carcinoma: a robust likelihood-based survival analysis
Source: Aging (Albany NY). 2020 Nov 5;12(21):21854–73. doi: 10.18632/aging.104001 (PMC7695399; doi:10.18632/aging.104001)
Supplement: Supplementary Table 6 [file aging-12-104001-s007..pdf]

SUPPLEMENTARY TABLE

Supplementary Table 6. Univariable cox regression of the 56 samples

Variable	HR	95% CI	P-value
Risk score (low risk vs high risk)	2.340	1.628-3.544	<0.001
Age (≤60 vs >60)	1.108	0.902-1.432	0.182
Stage (I and II vs III and IV)	2.422	1.135-4.551	0.033
Gender (male vs female)	2.052	1.207-8.678	0.028

CI: confidence interval; HR: hazard ratio.
